# ARNi: A Novel Approach to Counteract Cardiovascular Diseases

**DOI:** 10.3390/ijms20092092

**Published:** 2019-04-28

**Authors:** Massimo Volpe, Speranza Rubattu, Allegra Battistoni

**Affiliations:** 1 Department of Clinical and Molecular Medicine; School of Medicine and Psychology, Sapienza University of Rome, 00189 Rome, Italy; rubattu.speranza@neuromed.it (S.R.); alle.battistoni@gmail.com (A.B.); 2 IRCCS Neuromed, 86077 Pozzilli, Italy

**Keywords:** angiotensin receptor–neprilysin inhibitor, natriuretic peptides, renin–angiotensin system, heart failure, arterial hypertension

## Abstract

Cardiovascular diseases (CVDs) still represent the greatest burden on healthcare systems worldwide. Despite the enormous efforts over the last twenty years to limit the spread of cardiovascular risk factors, their prevalence is growing and control is still suboptimal. Therefore, the availability of new therapeutic tools that may interfere with different pathophysiological pathways to slow the establishment of clinical CVDs is important. Previously, the inhibition of neurohormonal systems, namely the renin–angiotensin–aldosterone system (RAAS) and the sympathetic nervous system, has proven to be useful in the treatment of many CVDs. Attempts have recently been made to target an additional hormonal system, that of the natriuretic peptides (NPs), which, when dysregulated, can also play a role in the development CVDs. Indeed, a new class of drug, the angiotensin receptor–neprilysin inhibitors (ARNi), has the ability to counteract the effects of angiotensin II as well as to increase the activity of NPs. ARNi have already been proven to be effective in the treatment of heart failure with reduced ejection fraction. New evidence has suggested that, in the next years, the field of ARNi application will widen to include other CVDs, such as heart failure, with preserved ejection fraction and hypertension.

## 1. Inhibition of Neurohormonal Systems in Cardiovascular Diseases

The worldwide prevalence of cardiovascular diseases (CVDs) is still alarmingly high. Although the CV mortality has declined between 1990 and 2015, mostly thanks to advantages conferred by interventional cardiology and anti-ischemic therapy, rates have plateaued in recent years, accounting for almost 18 million deaths/year in 2015 [1,2]. Accordingly, there were more than 422 million cases of CVDs/year globally, representing a major issue for healthcare systems [1,2]. Indeed, the prevalence of CVDs—and especially of congestive heart failure (HF), which is the final stage of different CVDs—has not yet started to decrease. The prognosis of patients affected by HF, both with preserved (HFpEF) and reduced ejection fraction (HFrEF), is still poor, highlighting the need for more effective preventive and treatment strategies [1].

Since the recognition of neurohormonal systems as being responsible for the development and progression of HF, neuroendocrine modulation with beta blockers targeting the sympathetic nervous system (SNS); angiotensin-converting enzyme inhibitors (ACEis), angiotensin receptor blockers (ARBs), and mineralocorticoid receptor antagonists (MRAs) for the renin–angiotensin–aldosterone system (RAAS) have been pivotal in the treatment of chronic HF and reducing associated morbidity and mortality. No additional effective pharmacotherapies have since been discovered [3]. A notable exception is the most recently discovered single molecule with a dual component, sacubitril/valsartan, which combines an ARB with the neutral endopeptidase inhibitor (NEPi) neprilysin, increasing the availability of natriuretic peptides (NPs) [4,5]. The development of this drug comes after decades of attempts to use NPs as therapeutic weapons in HF.

## 2. Natriuretic Peptides—Biological Properties

The NP family includes three different molecules—atrial natriuretic peptide (ANP), brain natriuretic peptide (BNP), and C-type natriuretic peptide (CNP)—that play a key role in cardiorenal homeostasis [6]. They are mostly synthesized within the heart in response to volume overload and myocyte stress, although synthesis in response to neuroendocrine regulation has also been shown [7,8]. Although they are synthesized as pre-prohormones their biologically active domain is the α-carboxy-terminal peptide [7,9]. NP effects are mediated by guanylyl cyclase (GC) receptors, with the NPR-A receptor being the main effector of both ANP and BNP, whereas the NPR-B receptor mediates CNP actions [7,9]. Both ANP and BNP contribute to regulating vascular tone, mainly due to their vasodilating properties. Indeed, ANP, through the activation of the cGMP-dependent protein kinase G (PKG), leads to increased production of nitric oxide (NO) with subsequent relaxation of the vascular smooth muscle cells and a decrease in blood pressure (BP) [10]. ANP may also induce Ca^2+^/calmodulin-dependent endothelial NO synthase in the aorta, ventricle, and kidney [11,12]. NPs also enhance diuresis and natriuresis, leading to lower BP levels. Indeed, ANP may enhance the glomerular filtration rate (GFR) through a direct vasodilating effect on the afferent arterioles with an increase in blood ultrafiltration. Moreover, ANP boosts natriuresis by acting directly on Na^+^ channels in the nephron, decreasing renin release from juxtaglomerular cells, and by inhibiting the synthesis and release of aldosterone [13,14]. NPs may also influence left ventricle afterload, not only acting on vascular tone and body fluid homeostasis, but also by decreasing adverse vascular remodeling over time. Indeed, high BNP levels may reduce the expression of transforming growth factor β (TGF-β), which is an important profibrotic molecule, and facilitate the degradation of extracellular fibrotic component [15,16,17]. In the heart, ANP counteracts the sympathetic nerve activity and increases vagal activity, leading to a decrease of heart rate and of cardiac output [18]. Moreover, NPs, by interacting with their G-coupled receptor, might antagonize pathological signaling leading to hypertrophy, such as cGMP-PKG deterioration, with titin hypophosphorylation [19], NO reduction with endothelial dysfunction [20], inflammation, and fibrosis [21].

Due to their multiple functions, the biological signature of NPs is to reduce body fluid and maintain BP and CV homeostasis. On this basis, NPs are physiological antagonists of both SNS and RAAS, and they are fundamental actors in HF.

## 3. Mechanisms of Degradation of NPs

The type C natriuretic peptide receptor (NPR-C) is mostly expressed in the kidney, adrenals, lungs, brain, heart, as well as vascular wall [22]. Unlike NPR-A and NPR-B, NPR-C exerts its biological action through receptor–ligand internalization, followed by lysosomal delivery of its ligand (NP) for degradation [23]. Therefore, NPR-C has been designated as the “clearance receptor”. Circulating NPs may also be cleared via proteolytic cleavage by neutral endopeptidase (NEP). Neprilysin is a membrane-bound zinc-dependent metallopeptidase acting on the amino side of hydrophobic residues. It is expressed in different tissues, including the myocardium, kidneys, brain, and vessels [24].

It has been shown that whenever levels of NPs are high, such as in HF, NEP becomes the main source for their metabolism [25]. The affinity of NEP for NPs depends, in part, on their ability to match their structure with the active site of NEP [26]. In this regard, ANP represents the major target of NEP action. Furthermore, amino-terminal NPs are not cleared by NEP [27].

NEP is responsible for the metabolism of more than 50 putative substrates. Indeed, NEP also accounts for the degradation of bradykinin, substance P, adrenomedullin, angiotensin II, and endothelin-1. As a result, its activity or, on the contrary, its blockade, may lead to complex effects [24]. Therefore, the benefits derived from NEP inhibition could be more that those related just to the increase in NP availability. In particular, the decrease in bradykinin metabolism may be important since high bradykinin levels enhance NO-mediated vasodilation and may modulate ischemic preconditioning [28,29,30]. On the other hand, it should also be noted that bradykinin, as well as substance P, may increase vascular permeability and, therefore, they may also be implicated in the development of angioedema [31], which is a potential side effect of NEP inhibition. NEP inhibition also increases adrenomedullin level. Proadrenomedullin may have a hypotensive effect by decreasing peripheral catecholamine release, increasing natriuresis and vasodilation through the cyclic adenosine monophosphate (cAMP), NO, and renal prostaglandin systems [32,33].

On the other hand, the inhibition of NEP increases angiotensin II and endothelin-1 levels which are both involved in vessel contraction and fibrosis, resulting in a consequent reduction of the beneficial effects derived from NP increase. Moreover, NEP converts angiotensin-I to angiotensin 1–7 [31] which conveys vasodilating, antiproliferative, and natriuretic effects through the activation of the Mas receptor [34]. Therefore, the inhibition of NEP will enhance substrate conversion to angiotensin-I, thereby potentiating the RAAS and neutralizing the advantages of NP augmentation [5,35,36]. This is the rationale for the need for a concomitant RAAS blockade. In the case of endothelin-1, NEP also hydrolyzes the big endothelin-1 precursor peptide. Thus, the effect of a NEP inhibitor on endothelin-1 levels will depend on the net effect of hydrolysis of both big-endothelin-1 and endothelin-1 [37]. The latter may lead to vasodilation through NO and prostaglandin by binding to the endothelin B receptor on endothelia. Conversely, binding to the endothelin A and endothelin B receptors on vascular smooth muscle cells induces vasoconstriction. In the myocardium, endothelin stimulates fibroblast synthesis of collagen and promotes cardiac hypertrophy [38,39].

Given its multiple substrates, the net effect deriving from the inhibition of NEP may be difficult to foresee as it catalyzes molecules with opposite effects on CV homeostasis.

## 4. Therapeutic Strategies Involving NP Metabolism in Cardiovascular Diseases

For therapeutic purposes, synthetic NPs that attempt to reproduce the beneficial effects of NPs were first developed. Among the synthetic peptides, anaritide and carperitide are synthetic forms of ANP, whereas nesiritide is a synthetic form of BNP [40]. Ularitide and cenderitide are the synthetic forms of urodilatin and CNP, respectively. These drugs have shown some benefits in the treatment of HF [9], but their effects are usually scant and their tolerability inadequate, so that their clinical use is not supported. On the other hand, inhibition of endogenous NP degradation has been attempted. Inhibition of NPR-C seemed unreliable because of its multiple functions other than NP clearance. The NEPi was first proposed for use in monotherapy. In the early 1990s, candoxatril was initially studied as an antihypertensive agent, but it was not found to have a sustained antihypertensive effect. It later became evident that the lack of an antihypertensive effect with NEPi alone was secondary to the increased levels of the vasoconstrictors such as angiotensin II and endothelin-1 [30,37,41]. Therefore, drugs combining a NEPi and an ACEi were developed [42]. However, these drugs were discharged because of the higher occurrence of angioedema that was dependent on the dual mechanism of action. Indeed, both ACE and NEP are enzymes responsible for the metabolism of bradykinin, which may cause vasodilation, angioedema, and airway obstruction.

Eventually, it was the time for a new class of medications, known as angiotensin receptor–neprilysin inhibitors (ARNi). Indeed, ARNi combine the NEP inhibition, due to sacubitril, with the ATII receptor I inhibition by valsartan (an ARB), offering the benefits of this two-step approach whilst avoiding the side effects of an increase in bradykinin due to dual inhibition of its metabolism. Indeed, the first-in-class ARNi, sacubitril/valsartan or LCZ696, contains valsartan and a NEPi prodrug, sacubitril (AHU377), in a 1:1 molar ratio. Upon ingestion, sacubitril is metabolized into an active NEPi, sacubitrilat (LBQ657). The target dose of 97/103 mg BID of sacubitril/valsartan resulted in equivalent plasma concentrations as valsartan 160 mg BID and a rise in cGMP, representing an increase in NPR activation, secondary to effective NEP inhibition [43]. Recent data suggest that the reduction in NEP activity results in a favorable impact of sacubitril/valsartan on HF progression, due especially to an increase in ANP and possibly CNP, rather than BNP [44].

## 5. Clinical Applications of ARNi

### 5.1. Heart Failure with Reduced Ejection Fraction

The prospective comparison of angiotensin receptor–neprilysin inhibitor with ACEi to determine impact on global mortality and morbidity in heart failure (PARADIGM-HF) trial first tested sacubitril/valsartan in HFrEF [5]. In PARADIGM-HF, sacubitril/valsartan was compared to enalapril in a cohort of patients affected by symptomatic HFrEF with left ventricular ejection fraction (LVEF) ≤ 35% and elevated B-type NP levels or hospitalization for HF within the previous year. Sacubitril/valsartan proved to be more effective than enalapril in reducing the primary outcome, a composite of death from CV causes or first hospitalization for HF (Table 1). Although there were more events of symptomatic hypotension in the case of using sacubitril/valsartan, more participants assigned to enalapril discontinued their study medication due to adverse effects. In clinical practice, this would mean a higher proportion of patients achieving optimal RAAS inhibition (as well as the additional benefits associated with concomitant neprilysin inhibition) with an ARNi rather than with enalapril. [5] Therefore, the latest American and European guidelines for the management of HF added sacubitril/valsartan as a first-line therapy for outpatients affected by chronic HFrEF [45,46].

Improvement in the prognosis of patients assigned to sacubitril/valsartan also remained consistent in the subgroup of prediabetic, undiagnosed diabetic, and diagnosed diabetic patients, who are at a higher risk of adverse CV outcomes [53]. This evidence agrees with previous preclinical data demonstrating the cardio- and nephroprotective effects of ARNi [54,55,56,57].

A subsequent analysis of the PARADIGM trial reported that sacubitril/valsartan use was associated with further evidence of clinical benefit in comparison with enalapril, including fewer visits to an emergency department for HF, a reduced need for intensification of the treatment for HF, and a lower requirement for intensive care, HF devices, or cardiac transplantation [47]. Moreover, another subsequent analysis of PARADIGM trial, which has enrolled almost half of the patients with a high CV risk, showed fewer coronary events in those treated with sacubitril/valsartan [58]. A recent experimental study in rats provided insight into the differential effects of sacubitril and valsartan in a model of HF. In particular, it has been shown that sacubitril in association with valsartan significantly improves load-dependent left ventricle contractility and relaxation with a reduction of myocardial collagen content, while the improvement in load-independent left ventricular contractility is due to valsartan [59].

Following the evidence for chronic HF, the PIONEER-HF study, a multicenter trial, has been designed to investigate the role of sacubitril/valsartan in patients affected by HFrEF hospitalized for an episode of acute HF (AHF), after hemodynamic stabilization, regardless of the duration of diagnosis or background HF therapy, and without a preceding run-in period. Thus, this trial has been performed in treatment-naïve hospitalized patients. The primary endpoint of PIONEER-HF was the proportional change in amino-terminal pro-brain natriuretic peptide (NTproBNP) level from baseline through one month and then two months. The main result was that sacubitril/valsartan led to a greater reduction in the NTproBNP concentration than enalapril from the first week of treatment, as well as to a decrease of markers of myocardial injury. Furthermore, in-hospital initiation of sacubitril/valsartan therapy was associated with a subsequent lower rate of rehospitalizations for HF. The rates of experienced side effects did not differ significantly between the sacubitril/valsartan group and the enalapril group [49].

More insights about the management of patients hospitalized for HF have been retrieved by the TRANSITION trial. This is a randomized, phase IV, multicenter, open-label study which assessed the safety and tolerability of introducing a therapy with sacubitril/valsartan in 1002 patients hospitalized for decompensated acute HFrEF still in the hospital or once discharged. Almost one-third of patients were newly diagnosed with HFrEF, and one-quarter were naïve to ACEi or ARB. The primary endpoint of achieving the target dose of sacubitril/valsartan 200 mg BID at 10 weeks after randomization has been achieved in 45% of patients that started taking sacubitril/valsartan in hospital, and in 50.4% of the post-discharge group, without any significant difference in adverse effects between the two groups [48].

Recently, subsequent analyses of previous trials have given more insightful data about a specific subset of patients. Indeed, a post hoc analysis of the PARADIGM trial investigated the effects of sacubitril/valsartan in diabetic patients, showing that this treatment leads to a better glycemic profile (reduction of Hb1Ac and less need to undertake insulin therapy or oral hypoglycemic agents) in the long term, independent of the reduction in body weight [60]. Similar beneficial effects of sacubitril/valsartan on lipid and glucose metabolism have also been reported in hypertensive obese patients as [61]. Preclinical models of diabetes seem to indicate that this beneficial effect of sacubitril/valsartan depends on the rise in NP levels, bradykinin, glucagon-like peptide 1, and on the reduction in angiotensin II levels that would result in subsequently improved insulin sensitivity [62,63]. These data may have great clinical relevance since diabetes is not only a comorbidity widely present among HF patients, but its evolution can substantially modify the patient prognosis. Moreover, recent interesting preclinical data seem to confirm the beneficial effects of sacubitril/valsartan on vascular and neural complications in type 2 diabetes, giving room for hypotheses about possible wider future applications of ARNi in diabetic patients [64].

### 5.2. Heart Failure with Preserved Ejection Fraction

Up to now, sacubitril/valsartan does not have an official indication in patients with HFpEF. However, it has also been supposed to play a beneficial effect in HFpEF by blocking a profibrotic/prohypertrophic mechanism (valsartan) while stimulating an antifibrotic/antihypertrophic mechanism (sacubitril) [65]. Indeed, the RAAS may play a pivotal role in enhancing the inflammation, endothelial dysfunction, and remodeling implicated in the progression of HFpEF. Despite this, RAAS inhibitors have failed to demonstrate mortality benefits in this setting. Beyond the single RAAS inhibition, the sacubitril/valsartan might ameliorate several key pathways in the development of HFpEF, namely cardiac remodeling, such as left ventricular hypertrophy and stiffness, microvascular dysfunction, and oxidative stress by increasing NPs levels [65]. Sacubitril/valsartan has been tested in a phase II trial in patients affected by HFpEF, the PARAMOUNT (prospective comparison of ARNi with ARB on management of heart failure with preserved ejection fraction) trial [66]. In this trial, sacubitril/valsartan 200 mg BID was compared with valsartan 160 mg BID in patients symptomatic for HFpEF, aged ≥40 years, with NTproBNP ≥400 pg/mL, while on diuretic therapy. The primary endpoint, the decline in NTproBNP at 12 weeks, was greater in the sacubitril/valsartan group. Furthermore, after 9 months, the left atrial dimension, which may indicate diastolic function [66], declined more in the sacubitril/valsartan arm as well as markers of fibrosis [51]. Subsequent analyses have shown that these sacubitril/valsartan effects were independent of the BP-lowering effect [67]. In addition, patients in the sacubitril/valsartan arm had greater improvements in New York Heart Association (NYHA) class and preserved better renal function compared to the valsartan group [68].

Given these favorable results, the current phase III PARAGON (prospective comparison of sacubitril/valsartan with ARB global outcome in HF with preserved ejection fraction) trial has been designed to determine whether sacubitril/valsartan can reduce CV death or total HF hospitalizations in patients with HFpEF. This trial has enrolled symptomatic patients with LVEF ≥45% and elevated NP, or history of HF hospitalization within 9 months and evidence of structural heart disease. The results of this trial are expected in 2019 [69].

Finally, the randomized, 24-week, double-blind multicenter controlled study comparing sacubitril/valsartan with medical therapy for comorbidities in HFpEF patients (PARALLAX) is currently recruiting participants to test the superiority of LCZ696 in reducing NTproBNP levels and improving HF symptoms and exercise function in HFpEF patients [70].

### 5.3. Hypertension

Preclinical studies have given insights on how may ARNi favorably exert antihypertensive and cardioprotective effects in animal models of hypertension [71]. In fact, a significant reduction of BP and proteinuria levels and a full prevention from stroke was observed over long-term treatment with sacubitril/valsartan, as compared to valsartan, in the high-salt-fed, stroke-prone, spontaneously hypertensive rat [71]. Furthermore, in a model of spontaneous hypertensive rat, sacubitril/valsartan proved to be as effective as valsartan in improving endothelium-dependent and -independent vasorelaxation [72]. Moreover, sacubitril/valsartan has shown an improved ability to reduce BP levels compared to valsartan, regardless of the amount of salt intake. This effect was associated with a significant increase of urinary sodium excretion and suppression of sympathetic activity. In addition, it reduced myocardial inflammation, remodeling, and endothelial dysfunction, also ameliorating coronary circulation [73].

In hypertensive patients, a proof-of-concept trial enrolling mostly white, mild-to-moderate hypertensive patients demonstrated that compared with valsartan or AHU377 alone, sacubitril/valsartan treatment for 2 months provided additional reduction of BP, systolic, diastolic, and pulse pressures, both sitting and ambulatory, without any excess in serious adverse effects [74]. In the PARAMETER (prospective comparison of angiotensin receptor–neprilysin inhibitor with angiotensin receptor blocker measuring arterial stiffness in the elderly) study, sacubitril/valsartan demonstrated efficacy in reducing arterial stiffness in the elderly with systolic hypertension and pulse pressure >60 mmHg [52]. At 3 months, sacubitril/valsartan reduced central aortic systolic BP more than olmesartan and reduced mean 24-hour ambulatory brachial and central aortic systolic BP, therefore, fewer patients in the sacubitril/valsartan group required add-on antihypertensives [52]. Similarly, a recent study in a cohort of elderly Asiatic patients affected by isolated systolic hypertension showed that sacubitril/valsartan was more effective than olmesartan in reducing mean systolic BP and pulse pressure [75]. Tolerability of sacubitril/valsartan and olmesartan was the same.

Furthermore, Ruilope and colleagues demonstrated that LCZ696 monotherapy was dose-dependently superior to valsartan monotherapy by clinical and ambulatory BP measurements for all tested doses [74]. Therefore, available evidence seems to support an application of ARNi as an antihypertensive compound with adequate tolerability and effectiveness throughout 24 hours.

## 6. Future Perspective of NP-Based Therapies

There are several ongoing studies to help understand ARNi doses and tolerability in different clinical settings, as well as to increase its possible fields of application.

Concerning HF, there are six ongoing trials investigating the possible benefit of ARNi on different endpoints: biomarker changes and ventricular remodeling among patients with HFrEF (PROVE-HF, NCT02887183) [76], changes in aortic impedance among patients affected by HF and hypertension (EVALUATE-HF, NCT02874794) [77], changes in functional mitral regurgitation (PRIME, NCT02687932) in patients with LVEF between 25% and 50% [78], and changes in mean pulmonary artery pressure in patients with reduced LVEF (PARENT, NCT02788656) [79]. The HFN-LIFE study (ClinicalTrials.gov Identifier: NCT02816736) will help to assess the safety and tolerability of the lowest dose of sacubitril/valsartan in patients with HFrEF symptomatic at rest [80]. Finally, the PARADOR (comparing ARNi with ACE inhibitor on endothelial function) trial is a multicenter, randomized, double-blind trial designed to compare the effects of sacubitril/valsartan vs. enalapril on endothelial function in patients with HFrEF [81].

Preliminary evidence in a mouse model of acute myocardial infarction (AMI) showed that LCZ696 significantly suppressed the production of proinflammatory cytokines, matrix metalloproteinase-9 activity, and aldosterone [82]. In a rat model, the association of sacubitril and valsartan has been found to protect myocardial ischemic damage by possibly ameliorating oxidative stress [83]. In addition, in two animal models, AMI sacubitril/valsartan has shown to determine the short- and long-term benefits in preventing MI-induced ventricular dysfunction compared to valsartan alone, with a reduction of fibrosis and myocardial scar and increased perfusion to the infarcted areas [84,85]. These findings provide a promising experimental basis to investigate the cardioprotective effects of sacubitril/valsartan in AMI patients. Therefore, a phase III, randomized, controlled PARADISE-AMI (prospective ARNi versus ACE inhibitor trial to determine superiority in reducing heart failure events after MI) study is currently recruiting post-AMI patients without prior HF, and with reduced LVEF or pulmonary congestion [86]. This trial will evaluate the benefit of sacubitril/valsartan versus ramipril in reducing the occurrence of the primary composite endpoint of CV death, HF hospitalization, and outpatient HF.

Lastly, preclinical data seem to support the hypothesis of a more beneficial effect of sacubitril/valsartan over valsartan alone in CV abnormalities associated with chronic kidney disease [87].

## 7. Conclusions

The most recent discovery in the field of CV therapy concerns the chance of interfering with NP metabolism. The development of the first drug used in clinical practice in this sense, thanks to its second active domain active as ARB, resulted in an improvement in mortality and morbidity in patients affected by HFrEF, for which the drug was primarily developed. It is also likely that deeper knowledge of NPs and NEP activity could soon make us reconsider the principles of therapy of many CVDs.

## Figures and Tables

**Table 1:** Main clinical trials about the effects of LCZ696 on cardiovascular outcomes.

Study; Aim	Study Population	Design	Outcomes
PARADIGM-HF (and post hoc analysis) Comparison of efficacy of LCZ696 versus enalapril in patients with HFrEF (LVEF ≤35%) [5,47]	Patients with symptomatic HFrEF (NYHA functional classes II to IV), and elevated B-type natriuretic peptide levels or hospitalization for HF within the previous 12 months; *n* = 8442	Multicenter, randomized, double-blind study	LCZ696 reduced the composite primary of CV death or HF hospitalization more than enalapril; LCZ696 reduced secondary endpoint more than enalapril: • any CV death; • first worsening HF hospitalization; • all-cause mortality Moreover, LCZ696 group had fewer hospitalizations for worsening HF, less necessity to receive intensive care, intravenous positive inotropic agents, and to have implantation of a HF device or cardiac transplantation.
TRANSITION To assess the safety and tolerability of starting a therapy with LCZ696 while still in the hospital or after discharge [48]	HFrEF patients hospitalized for ADHF, after stabilization *n* = 1002	Multicenter, randomized, open-label, parallel-group study	The percentage of patients taking target dose of sacubitril/valsartan 200 mg BID at 10 weeks post randomization was the same among patients who started taking LCZ696 during hospitalization or after discharge
PIONEER-HF To assess the percentage change from baseline in NTproBNP levels with LCZ696 [49]	HFrEF patients hospitalized for ADHF after stabilization *n* = 736	Multicenter, randomized, double-blind study	LCZ696 led to a reduction in the NTproBNP concentration than a therapy with enalapril at 4 and 8 weeks; LCZ696 led to a reduction in the level of high-sensitivity cardiac troponin T; LCZ696 led to lower rate of rehospitalization for HF
TITRATION To assess the tolerability of initiating/uptitrating LCZ696 from 50 to 200 mg BID over 3 and 6 weeks [50]	Patients with symptomatic HFrEF (NYHA functional classes II to IV) + one or more of the following additional eligibility requirements: for outpatients currently treated with ACEi/ARB, the dose must have been stable for at least 2 weeks; to be classified as ACEi/ARB-naïve, the patient must not have taken ACEi/ARB for at least 4weeks; hospitalized patients had to be either ACEi/ARB-naïve, or on a tolerated dose of an ACEi/ARB at screening *n* = 429	Multicenter, randomized, double bind, parallel study	Initiation/uptitration of LCZ696 from 50 to 200 mg BID had a tolerability profile in line with other HF treatments.
PARAMOUNT To assess the efficacy of LCZ96 versus valsartan to change NTproBNP levels from baseline [51]	Patients with signs and symptoms of HF, ≥40 years, with NTproBNP ≥400 pg/mL and a LVEF ≥45%, while on active diuretic therapy *n* = 301	Multicenter, randomized, double-blind study	The decline in NTproBNP at 12 weeks after initiation of the treatment was greater in the LCZ696 group. LCZ969 was also able to ameliorate LA size and NHYA class (secondary endpoints)
PARAMETER To assess the efficacy of LCZ696 versus olmesartan in reducing arterial stiffness [52]	Elderly patients (aged ≥60 years) with systolic hypertension and pulse pressure >60 mmHg *n* = 454	Multicenter, randomized, double-blind study	LCZ696 reduced central aortic SBP more than olmesartan and reduced mean 24-hour ambulatory brachial and central aortic SBP

ACEi: angiotensin converting enzyme inhibitors; ARB: angiotensin II receptor I blockers; CV: cardiovascular; ADHF: acute decompensated heart failure; BID: bis in die; LVEF: left ventricular ejection fraction; HFrEF: heart failure with reduced ejection fraction; HFrpEF: heart failure with preserved ejection fraction; NTproBNP: amino-terminal pro-brain natriuretic peptide; NYHA: New York Heart Association; SBP: systolic blood pressure.
